# Complete Genome Sequence of Cyanobacterium *Geminocystis* sp. Strain NIES-3709, Which Harbors a Phycoerythrin-Rich Phycobilisome

**DOI:** 10.1128/genomeA.00385-15

**Published:** 2015-04-30

**Authors:** Yuu Hirose, Mitsunori Katayama, Yoshiyuki Ohtsubo, Naomi Misawa, Erica Iioka, Wataru Suda, Kenshiro Oshima, Mitsumasa Hanaoka, Kan Tanaka, Toshihiko Eki, Masahiko Ikeuchi, Yo Kikuchi, Makoto Ishida, Masahira Hattori

**Affiliations:** a Department of Environmental and Life Sciences, Toyohashi University of Technology, Tempaku, Toyohashi, Aichi, Japan; b Electronics-Inspired Interdisciplinary Research Institute (EIIRIS), Toyohashi University of Technology, Tempaku, Toyohashi, Aichi, Japan; c College of Industrial Technology, Nihon University, Narashino, Chiba, Japan; d Department of Environmental Life Sciences, Graduate School of Life Sciences, Tohoku University, Sendai, Miyagi, Japan; e Center for Omics and Bioinformatics, Graduate School of Frontier Sciences, The University of Tokyo, Kashiwa, Chiba, Japan; f Department of Microbiology and Immunology, Keio University School of Medicine, Shinjuku-ku, Tokyo, Japan; g Division of Applied Biological Chemistry, Graduate School of Horticulture, Chiba University, Matsudo, Chiba, Japan; h Chemical Resources Laboratory, Tokyo Institute of Technology, Midori-ku, Yokohama, Kanagawa, Japan; i Department of Life Sciences (Biology), The University of Tokyo, Meguro, Tokyo, Japan; j Graduate School of Advanced Science and Engineering, Waseda University, Shinjuku-ku, Tokyo, Japan

## Abstract

The cyanobacterium *Geminocystis* sp. strain NIES-3709 accumulates a larger amount of phycoerythrin than the related NIES-3708 strain does. Here, we determined the complete genome sequence of the NIES-3709 strain. Our genome data suggest that the different copy number of rod linker genes for phycoerythrin leads to the different phycoerythrin contents between the two strains.

## GENOME ANNOUNCEMENT

Certain cyanobacteria species modulate the composition of light-harvesting antenna proteins, phycoerythrin and phycocyanin, within the phycobilisome. This phenomenon is called complementary chromatic acclimation (CCA) (1, 2) and is conventionally classified as two types (3): type II species that modulate phycoerythrin content only, and type III species that modulate both phycoerythrin and phycocyanin content. Recent studies showed that type II species utilize the CcaS-CcaR photosensory system for CCA (4, 5), whereas type III species utilize the RcaE-RcaF-RcaC system (6–9). In the type II CCA, the CcaS-CcaR system directly regulates the expression of the rod linker gene of phycoerythrin and, in several species, the hydrophobic rod-core linker of phycocyanin (10).

The cyanobacterium *Geminocystis* sp. strain NIES-3709 accumulates a larger amount of phycoerythrin than the related NIES-3708 strain does, although the two strains are isolated from the same freshwater stream. We already reported the complete genome sequence of the NIES-3708 strain. To explore the molecular basis of the different cellular phycoerythrin contents in the two strains, we performed whole-genome sequencing of the NIES-3709 strain using the MiSeq (Illumina) system. An 800-bp paired-end library and an 8-kbp mate-pair library were prepared using the TruSeq DNA PCR-free sample preparation kit (Illumina) and Nextera mate-pair sample preparation kit (Illumina), respectively. The libraries were sequenced on the MiSeq instrument with the MiSeq reagent kit version 3 (600 cycles; Illumina). The reads were filtered using ShortReadManager, based on a 17-mer frequency (11). A total of eight million paired-end reads (209 Mbp) and 10 million mate-pair reads (150 Mbp) were assembled using Newbler version 2.8 (Roche), yielding 11 scaffolds and 156 large contigs (>1 kbp). The sequence gaps between the contigs were determined *in silico* using GenoFinisher and AceFileViewer (11). We succeeded in determining the complete genome sequence of *Geminocystis* sp. NIES-3709, which comprises one chromosome and 12 plasmids (total, 4,426,059 bp). The G+C content of the genome was calculated to be 33%. A total of 3,937 protein-coding genes, 6 rRNA genes, and 44 tRNA genes were predicted using the Rapid Annotations using Subsystems Technology (RAST) (12).

The CCA genes of the NIES-3709 strain consist of a CcaS-CcaR photosensory system and a putative light-regulated *cpeE-cpeR* operon, which is the same structure of the CCA genes of the NIES-3708 strain. The NIES-3709 strain harbors single copies of genes of the rod-core linker of phycocyanin (*cpcG*), core of phycocyanin (*cpcB* and *cpcA*), and core of phycoerythrin (*cpeB* and *cpeA*), whose copy numbers are also the same as those of the NIES-3708 strain. However, we found that the total copy number of rod linker genes of phycoerythrin (*cpeC* and *cpeE*) of the NIES-3709 strain is four, whereas that of the NIES-3708 strain is three. This difference may reflect the different rod structure of phycobilisome in the two strains that leads the different cellular phycoerythrin contents. Further biochemical analysis is required to explore this hypothesis.

### Nucleotide sequence accession numbers.

The complete genome sequence of *Geminocystis* sp. NIES-3709 has been deposited in the DNA Data Bank of Japan under accession numbers AP014821 through AP014832.
